# Why is Mortalin a Potential Therapeutic Target for Cancer?

**DOI:** 10.3389/fcell.2022.914540

**Published:** 2022-06-29

**Authors:** A-Rum Yoon, Renu Wadhwa, Sunil C Kaul, Chae-Ok Yun

**Affiliations:** ^1^ Department of Bioengineering, College of Engineering, Hanyang University, Seoul, South Korea; ^2^ Institute of Nano Science and Technology (INST), Hanyang University, Seoul, South Korea; ^3^ Hanyang Institute of Bioscience and Biotechnology (HY-IBB), Hanyang University, Seoul, South Korea; ^4^ AIST-INDIA DAILAB, National Institute of Advanced Industrial Science and Technology (AIST), Tsukuba, Japan; ^5^ GeneMedicine CO, Ltd, Seoul, South Korea

**Keywords:** mortalin, cancer, withaferin A, withanone, MKT-077, mortaparib, i-mot ab, mot-Adon

## Abstract

Cancer is one of the leading causes of death worldwide, accounting for nearly 10 million deaths in 2020. Therefore, cancer therapy is a priority research field to explore the biology of the disease and identify novel targets for the development of better treatment strategies. Mortalin is a member of the heat shock 70 kDa protein family. It is enriched in several types of cancer and contributes to carcinogenesis in various ways, including inactivation of the tumor suppressor p53, deregulation of apoptosis, induction of epithelial–mesenchymal transition, and enhancement of cancer stemness. It has been studied extensively as a therapeutic target for cancer treatment, and several types of anti-mortalin molecules have been discovered that effectively suppress the tumor cell growth. In this review, we 1) provide a comprehensive sketch of the role of mortalin in tumor biology; 2) discuss various anti-mortalin molecules, including natural compounds, synthetic small molecules, peptides, antibodies, and nucleic acids, that have shown potential for cancer treatment in laboratory studies; and 3) provide future perspectives in cancer treatment.

## Introduction

Mortalin is a heat-uninducible member of the heat shock 70 kDa (HSP70) protein family. Although it is not a heat-activated protein, it is identified as a member of HSP70 because of its sequence similarity. Mortalin is located in the mitochondria, endoplasmic reticulum, plasma membrane, cytoplasmic vesicles, and cytosol and exhibits a differential subcellular distribution pattern in normal and transformed human cells (Ran et al., 2000; Wadhwa et al., 2002). It was initially cloned as a pan-cytosolic mortalin-1/mortality factor (mot-1) that induced senescence in immortalized mouse fibroblasts (Wadhwa et al., 1993a; Wadhwa et al., 1993b). Perinuclear mortalin (mot-2), which differs from mot-1 by two amino acids at the C-terminus (V618M and R624G), was identified later. In contrast to mot-1, it causes malignant transformation of immortalized mouse fibroblasts and enhances the longevity of human fibroblasts *in vitro* (Kaul et al., 1998; Yokoyama et al., 2002; Kaul et al., 2003). In contrast to mouse mortalin (mot-1 and mot-2), human cells were shown to possess only one kind of mortalin that was found to possess transforming activity similar to that of mouse mot-2 and was thus called hmot-2. Based on its subcellular localization, mortalin interacts with multiple proteins, including tumor suppressor p53, fibroblast growth factor-1, interleukin-1 receptor type 1, glucose-regulated protein 94, translocase of inner mitochondrial membrane (TIM)-44, and TIM23 (Kaul S et al., 2007) and is associated with diverse molecular pathways.

Through several signaling pathways, mortalin induces proliferation, migration, stemness, epithelial–mesenchymal transition, and angiogenesis in cancer cells (Wadhwa et al., 2006; Kaul S et al., 2007; Rozenberg et al., 2013; Na et al., 2016; Yun et al., 2017). Mortalin sequesters p53 in the cytosol of cancer cells and inhibits its translocation to the nucleus, thus hampering several functions of p53, including transcriptional activation, centrosome duplication, and apoptosis in cancer cells (Wadhwa et al., 1998; Ma et al., 2006; Lu et al., 2011a; Lu et al., 2011b; Yang et al., 2011). Furthermore, it increases Ras activity and regulates the Raf/mitogen-activated protein kinase (MEK)/extracellular signal-regulated kinase (ERK) pathway to promote the proliferation of cancer cells and protect them from apoptosis (Ichihara et al., 2004; Wu et al., 2013). Mortalin can also enhance the activities of telomerase and heterogeneous ribonucleoprotein K (hnRNP-K), which are related to the malignant transformation of human cancer cells (Ryu et al., 2014). It is associated with an increase in the expression levels of mesenchymal markers and downregulation of the expression levels of epithelial markers, resulting in increased stemness and metastasis of cancer cells (Na et al., 2016; Yun et al., 2017).

Based on the above findings, mortalin has been investigated as a potential therapeutic target in various modalities, such as natural compounds, chemical compounds, nucleic acids, peptides, and antibodies, for the treatment of cancer. This review examines the molecular mechanisms underlying the role of mortalin in carcinogenesis, explores current strategies to target mortalin for the treatment of cancer in preclinical stages, and discusses future perspectives and directions for the successful application of mortalin-targeted therapeutics in the clinical setting.

## Role of Mortalin as a Therapeutic Target

Mortalin is a highly upregulated protein in various cell types and tumor tissues of patients with cancer (Wadhwa et al., 2006). In laboratory studies, the overexpression of mortalin in cancer cells enhances their malignant properties, such as increased proliferation, migration, and invasion, via various cancer signaling pathways. Wadhwa et al. (1998) demonstrated a novel mechanism of p53 inactivation by mortalin in cancer cells (Wadhwa et al., 1998). They showed that perinuclear-distributed mortalin (hmot-2) binds to p53 in the cytoplasm and nucleus of human cancer cells, thereby inactivating the tumor suppressor activities of p53. Notably, mortalin and p53 are colocalized in immortalized and malignantly transformed cells, whereas normal cells do not show such colocalization of the two proteins, accounting for selective targeting of these proteins for cancer therapy (Ichihara et al., 2004; Lu et al., 2011a; Lu et al., 2011b). *In vitro* reporter assays of p53 revealed that the exogenous expression of hmot-2 downregulates the transcriptional activation of p53, as determined by p53-responsive genes (p21^WAF−1^ and hdm-2). Mortalin directly binds to p53 and inhibits its nuclear translocation, which leads to its transcriptional activation. Molecular analysis revealed the mortalin and p53-binding domains, wherein N-terminal amino acid residues (325–355) of mortalin bind to the C-terminal amino acid residues (355–393) of p53 (Iosefson and Azem, 2010). Ma et al. further identified that mortalin is specifically associated with duplicated centrosomes, which is reported in most human cancer types and is a major cause of chromosome instability in cancer cells (Ma et al., 2006; Denu et al., 2016). Excessively expressed mortalin reverses the p53-dependent suppression of centrosome duplication, as binding of mortalin and p53 can dsirupt the interaction between p53 and centrosome. Mortalin is involved in the uncontrolled duplication of the centrosome, which is a hallmark of cancer. Interestingly, mortalin itself is localized in the nuclei of cancer cells, where it contributes to increased proliferation and oxidative stress tolerance in cancer cells (Ryu et al., 2014). Ryu et al. have shown that cancer cells expressing a mortalin mutant, which lacks a mitochondrial-targeting signal peptide and is largely localized in the nucleus (hmot-N), exhibits tolerance against oxidative stress and increased malignant properties. hmot-N protects the cancer cells against endogenous and exogenous oxidative stress. Furthermore, hmot-N-expressing cells show a rapid proliferation rate, colony forming efficacy, motility, and tumor forming capacity *in vitro*, which is also translated to increased tumor growth and aggressive metastasis in mouse tumor models. Nuclear mortalin activates telomerase and hnRNP-K proteins, thereby contributing to the malignant phenotype of cancer cells (Abdullah et al., 2015).

Mutations in p53 are the most common genetic events that promote carcinogenesis. Several mutants exhibit growth arrest and apoptotic activities. However, several other factors, such as binding to other proteins, cause the inactivation of these functions of mutant p53 forms (Mantovani et al., 2019). In this context, the binding of mortalin to mutant forms of p53 was examined, and it was reported that mortalin inhibited p53-mediated tumor cell-specific apoptosis in cells harboring both wild-type and mutant p53 proteins. Downregulation of mortalin in these cell types activates p53 pathways and causes either growth arrest or apoptosis in cancer cells (Lu et al., 2011a; Lu et al., 2011b). Mortalin activates the phosphatidylinositol 3-kinase (PI3K)/AKT pathway and facilitates its crosstalk with the Raf/MEK/ERK pathway (Yang et al., 2011). Mortalin-mediated activation of the PI3K/AKT pathway can suppress the conformational changes in the Bcl-2-associated X (Bax) protein and block cytochrome c release, ultimately inhibiting mitochondria-mediated apoptosis. Mortalin regulates the ERK-mediated transcriptional activation of hypoxia inducible factor-1α (HIF-1α) (Mylonis et al., 2017). When ERK is inactivated, mortalin binds to HIF-1α and associates with voltage dependent anion channel 1 (VDAC1) and hexokinase II, which are located in the outer mitochondrial membrane (Mylonis et al., 2017). This complex (mortalin–VDAC1–hexokinase II–HIF-1α) confers resistance to apoptosis in cancer cells. The correlations among mortalin expression levels, carcinogenesis, metastatic potential, and tumor recurrence have been well documented. The Cancer Genome Atlas (TCCA) and Human Protein Atlas analyses revealed that cancer cells acquiring metastatic potential are closely correlated with the expression levels of mortalin (Na et al., 2016). Mortalin/HSP9A gene locus and mRNA expression were frequently amplified in cancer patients; upregulation of mRNA was most frequent. Interestingly, human protein atlas indicates that the level of HSPA9 (mortalin) can be unfavorable (breast cancer) or favorable (colorectal cancer). The P score, which demonstrate correlation of mRNA expression level and patient survival rate, is higher in the population of colorectal cancer patient (P score: 0.00045) than that of breast cancer patient (P score: 0.00029). Further, the overexpression of mortalin causes an increase in the migration and invasion of cancer cells via upregulation of the expression levels of proteins, such as focal adhesion proteins, as well as the PI3K-Akt and JAK-STAT signaling pathways. Mortalin enhances the expression levels of mesenchymal markers (vimentin, fibronectin, and β-catenin), while downregulating the expression levels of epithelial markers (E-cadherin, CK8, and CK18), contributing to epithelial–mesenchymal transition and cancer metastasis. Moreover, mortalin overexpression causes an increase in cancer cell stemness by enhancing the expression levels of stem cell markers, such as ATP binding cassette subfamily G member 2 (ABCG2), POU class 5 homeobox 1 (POU5F1/OCT-4), CD133, aldehyde dehydrogenase 1 (ALDH1), CD9, ATP binding cassette subfamily C member 1 (ABCC1/MRP1), and connexin (Yun et al., 2017). It also caused drug resistance in cancer cells and ability to form spheroids.

Thus, mortalin promotes cancer activities via multiple pathways. These include binding to p53, inhibiting its nuclear localization and transcriptional activation, inhibition of centrosome duplication, and apoptosis. Based on the extensive findings described above, mortalin has been considered a promising candidate target for the development of anticancer drugs. Several therapeutic platforms targeting mortalin at the preclinical stage are described in the following sections.

## Therapeutic Platforms for Targeting Cancer

### Natural Compounds

The most current standard therapies for cancer are chemotherapeutic drugs. However, chemotherapy can cause severe adverse effects. Therefore, the development of new natural compounds is necessary to control the exponentially increasing incidence of cancer worldwide. The traditional natural anticancer compound that targets mortalin is the extract of Ashwaganda (*Withania somnifera*: Solanaceae). Ashwagandha are steroidal alkaloids and lactones, a class of chemicals known as withanolides (Kulkarni and Dhir, 2008). Withaferin A (Wi-A) and Withanone (Wi-N) are structurally similar to anolides isolated from Ashwagandha. Wi-A induces apoptosis (Adams et al., 2002; Malik et al., 2007; Srinivasan et al., 2007; Yang et al., 2007), and inhibits Notch-1 signaling exerting downregulation of Akt/nuclear factor-kB/Bcl-2 pathways (Koduru et al., 2010). It further suppresses inflammation by inhibiting NO production and inducible nitric oxide synthase expression by blocking Akt and NF-kappa B activity (Oh et al., 2008). Wi-A inhibits chymotrypsin like proteasomal activity, protein kinase C, and Akt and Raf-1 pathways, leading to tumor suppression by induction of apoptosis and cell adhesion (Adams et al., 2002; Sen et al., 2007). Wi-A and Wi-N showed different activities in human normal and cancer cells (Vaishnavi et al., 2012). Wi-A showed strong cytotoxicity in both cancer and normal cells. It showed significant anti-migratory, anti-invasive, and anti-angiogenic activities in both *in vitro* and *in vivo* assays (Yang et al., 2012; Kim et al., 2016). Wi-N, however, showed milder cytotoxicity and selective killing of cancer cells. Through combination treatment of Wi-N with Wi-A, the cytotoxicity of Wi-A was reduced in normal cells, while retaining their anticancer potential in cancer cells (Gao et al., 2014). Furthermore, combination of Cucurbitacin with Wi-N could induce senescence in cancer cells, leading to inhibition of tumor growth in A549 tumor xenograft model (Bhargava et al., 2019). Ashwagandha alcoholic leaf extract (i-Extract), enriched in Wi-N, was shown to selectively kill cancer cells and interact with p53, hmot-2, p21^WAF1^, and Nrf2 (Sen et al., 2007; Widodo et al., 2008; Widodo et al., 2010). Especially, there are at least five different pathways involved in i-Extract-mediated kill cancer cells, including p53 signaling, GM-CFS signaling, death receptor signaling, apoptosis signaling and G2-M DNA damage regulation pathway (Widodo et al., 2008). The i-Extract, fraction F1, fraction F4 and i-Factor caused an abrogation of mortalin-p53 interactions and reactivation of p53 function while the fractions F2, F3, F5 work through other mechanisms. Further, the results of bioinformatics revealed that i-Extract-mediated cancer cell killing was closely linked with the ROS signaling and induction of oxidative stress (Widodo et al., 2010). Collectively, these results demonstrated that i-Extract and Wi-N could enhance anti-tumor effect via both p53-dependent and -independent pathways.

Caffeic acid phenethyl ester (CAPE; C_1_7H_16_O_4_), a key bioactive ingredient of New Zealand honeybee propolis, can disrupt the mortalin–p53 complex, leading to the translocation and activation of p53 in the nucleus (Li et al., 2010; Wadhwa et al., 2016). Therefore, it can activate DNA damage signaling in cancer cells. CAPE can also be combined with Wi-A that is cytotoxic to both cancer and normal cells (Sari et al., 2020). Wi-A and CAPE cause inactivation of PARP-1-mediated DNA repair, resulting in accumulation of DNA damage and activation of apoptosis signaling. Although CAPE has been utilized in monotherapy or combination therapy, it is not stable in culture medium and is easily degraded into caffeic acid (C_9_H_8_O_4_) by secreted esterase, making it unsuitable for cancer treatment. To this end, Ishida et al. complexed CAPE with γ-cyclodextrin (γCD) to enhance the activity of CAPE (Ishida et al., 2018). The γCD-complexed CAPE showed high efficacy in anti-tumor and anti-metastasis assays *in vitro* and *in vivo.* Furthermore, cytotoxicity of the CAPE-γCD complex to a wide range of cancer cells is stable in an acidic milieu, showing that it can be a potent anticancer agent.

Similar to CAPE, artepillin C (ARC) from Brazilian green propolis docks into and abrogates mortalin-p53 complexes through multiple hydrogen bonds and hydrophobic interactions, causing the activation of p53 and growth arrest of cancer cells (Bhargava et al., 2018). ARC and green propolis-supercritical extract (GPSE) demonstrated high cytotoxicity *in vitro*. GPSE-conjugated γCD (GPSE-γCD) exhibited more potent anticancer activity than purified ARC. These data suggest that the bioactive ingredient of propolis can be used as a natural efficient and welfare anti-tumor composite.

Abdullah et al. reported that veratridine (VTD), an alkaloid derived from the Liliaceae plant, exerts potent cancer chemosensitivity via UBX domain protein 2A (UBXN2A)-dependent inhibition of mortalin (Abdullah et al., 2015). VTD enhances the transactivation of cytoplasmic UBXN2A, where UBXN2A binds and inhibits mot-2 oncoprotein. The combination of VTD with a suboptimal dose of the standard chemotherapy, 5-Fluorouracil (5-FU), and etoposide demonstrated a synergistic effect. UBXN2A and cytoplasmic mot-2 protein levels are low in tumor tissues; thus, VTD could enhance tumor-specific toxicity, while normal cells remain intact. Furthermore, VTD or its modified analogs offer a valuable adjuvant chemotherapy strategy to improve the efficacy of 5-FU-based chemotherapy in colon cancer patients harboring WT-p53. Therefore, VTD-mediated UBXN2A expression may serve as a novel target for colon cancer treatment.

Targeting mortalin *via* Embelin, a natural quinone, could increase the nuclear translocation of p53, leading to growth arrest of cancer cells (Nigam et al., 2015). Bioinformatics and molecular docking analyses demonstrated that binding of embelin to the mortalin/p53 complex abolishes the complex, resulting in nuclear translocation and transcriptional activation of p53. Furthermore, embelin-treated cells showed downregulation of growth factors and metastatic signaling pathways, indicating that embelin could be a promising therapeutic target of mortalin for the treatment of cancer metastasis.

Fucoxanthin, found in marine organisms, suppresses the transcriptional activity of mortalin, thus activating p53 function in cancer cells (Wang et al., 2014; Garg et al., 2019). As a result, fucoxanthin-mediated downregulation of mortalin caused a decrease in the hallmark proteins associated with cell proliferation and survival. The migration and invasion of cancer cells was also decreased by treatment of fucoxanthin. Of note, the anti-growth and anti-migration effects of Fucoxanthins on cancer cells are irrespective of the p53 status of cancer cells, indicating that mortalin is one of the targets among various fucoxanthin’s target genes (Garg et al., 2019). Furthermore, fucoxanthin is not toxic to normal cells, demonstrating its cancer cell-specific killing effects. These data suggest that the cancer therapy regimen may benefit from the recruitment of fucoxanthin; hence, it warrants further attention for basic mechanistic studies and drug development.

### Chemical Compounds

MKT-077 (C_21_H_22_ClN_3_OS_2_) is a classic chemical inhibitor of mortalin, which functions by eliminating mortalin–p53 interactions but does not alter the expression of mortalin (Miyata et al., 2013). MKT-077 exhibits significant anti-tumor activity in a variety of *in vitro* and *in vivo* (Koya et al., 1996; Tikoo et al., 1999; Tikoo et al., 2000). It binds to mot-2 and abrogates its interaction with the tumor suppressor protein p53. In cancer cells, but not in normal cells, MKT-077 induces the release of wild-type p53 from p53-mot-2 complexes and rescues its transcriptional activation (Wadhwa et al., 2000). MKT-077 could further overcome the limitations of photodynamic therapy (PDT), such as low specificity and resistance, which are mainly associated with oxidative stress. The cancer cell viability was permanently reduced by the MKT-077 in a dose-dependent manner by inducing apoptosis or necrosis, mainly under oxidative stress conditions. MKT-077-mediated inhibition of mortalin could also cause a decrease in drug resistance in cancer cells.

Mortaparib was identified by screening a chemical library of compounds that have the potential to abrogate cancer cell-specific mortalin-p53 interactions. It exerts p53 enrichment in the nucleus and shifts mortalin from perinuclear to pan-cytoplasmic. Intriguingly, it targets mortalin, poly ADP-ribose polymerase-1 (PARP-1), and mortalin-PARP-1 interactions, resulting in PARP-1 inhibition that triggers growth arrest/apoptosis signaling. Mortaparib-treated cells showed inhibition of cancer cell migration, metastasis, and angiogenesis *in vitro*. More importantly, mortaparib demonstrated potent anti-tumor and anti-metastatic effects in tumor xenograft models. This research marked the discovery of mortaparib as the first dual inhibitor of mortalin and PARP-1, exhibiting its molecular mechanism of action and demonstrating *in vitro* and *in vivo* tumor suppressor activity, ultimately emphasizing its potential as an anticancer drug (Putri et al., 2019). A second member of the Mortaparib class of mortalin inhibitors was also isolated from the same screening. It was identified as a novel synthetic small-molecule triazole derivative (4-[(1E)-2-(2-phenylindol-3-yl)-1-azavinyl]-1,2,4-triazole) and named Mortaparib^Plus^. Bioinformatics and computational analyses predicted that Mortaparib^Plus^ can competitively prevent the interaction of mortalin with p53, as it interacts with the p53 binding site of mortalin. Mortaparib^Plus^, mediated by the activation of p21WAF1 or BAX and PUMA signaling, induces growth arrest and apoptosis, respectively (Elwakeel et al., 2021). Furthermore, Mortaparib^Plus-^induced cancer cell death occurs through multiple mechanisms, including the inhibition of PARP-1, upregulation of p73, and the downregulation of mortalin and CARF proteins that play critical roles in carcinogenesis.

SHetA2 (NSC 726189), a small-molecule drug that disrupts mortalin/p53 complexes in ovarian cancer cells (Ramraj et al., 2020), is currently being developed for clinical applications based on its induction of apoptosis in cancer cells, whereas its effect on healthy cells is limited to G1 cell cycle arrest (Guruswamy et al., 2001; Liu et al., 2009; Masamha and Benbrook, 2009). Its methylated analog, PRIMA-1MET (APR-246), exhibited cytotoxicity and synergy with chemotherapy in cancer cell lines and is undergoing clinical investigation (Bykov et al., 2005). For the treatment of high-grade serous ovarian cancer (HGSOC), which has TP53 gene mutations with 96–100% frequency, PRIMA-1MET, a p53 reactivator that modifies amino acids in the mutant p53 core domain, was combined with SHetA2. In the orthotopic ovarian tumor model, untreated, SHetA2-, and PRIMA-1MET-treated mice showed tumor-free rates of 0, 25, and 42%, respectively. Interestingly, a 67% tumor-free rate was observed in the combination treatment with SHetA2 and PRIMA-1MET, indicating its ability to prevent tumors. This study demonstrated that dual targeting mortalin/p53 complexes and mutant p53 can be an alternative treatment strategy for HGSOC.

### Peptides

Nef is secreted from infected cells in exosomes and is abundant in the serum of HIV-infected individuals. The secretion modification region (SMR; amino acids 66–70) of Nef, which is required for secreted exosomal Nef, induces apoptosis in uninfected CD4 + T cells (Shelton et al., 2012). SMR-derived peptides can block extracellular vesicle secretion and mediate cell cycle arrest in MDA-MB-231 and MCF-7 breast cancer cells. Notably, this peptide was found to disrupt the interaction of HIV-1 Nef with mortalin. Based on this finding, a series of peptides derived from SMR was developed by adding a polyethylene glycol (PEG)-complexed clusterin-binding peptide (CLU). Exposure to SMR-CLU antagonized the functions of mortalin, blocking tumor extracellular vesicle release and extracellular vesicle-mediated release of complement. This leads to a decrease in breast cancer cell metastasis and allows the standard treatment of these late-stage tumor cells, thus having important clinical implications for breast cancer chemotherapy. Furthermore, PEG-SMRwt-CLU peptides inhibited the growth of human breast cancer cells and blocked tumor exosome release *in vitro* (Huang et al., 2017). The peptide alone did not cause increased cytotoxicity or apoptosis induction, but it did cause cell cycle G2/M phase arrest in both estrogen-responsive and non-responsive breast cancer cells. Furthermore, the SMRwt peptides increased the sensitivity of breast cancer cells to cisplatin and paclitaxel. These data suggest a potential therapeutic value of SMR in preventing breast cancer metastasis and as an adjuvant for the chemotherapeutic treatment of human breast cancer.

Interestingly, two mortalin-mimetic peptides (Mot-P2 and Mot-P7) with amino acid sequences predicted to be involved in the interactions of mortalin were designed to induce cytotoxicity in malignant cells (Jubran et al., 2020). Both Mot-P2 and Mot-P7 significantly enhanced antibody-mediated and complement-dependent cell killing in peptide-treated cancer cells. Mot-P2 and Mot-P7 increase necrotic cell death, leading to plasma membrane perforation, mitochondrial inner membrane depolarization, and decreased ATP levels. Furthermore, the combination of rituximab-mediated complement-dependent cytotoxicity with Mot-P2 or Mot-P7 resulted in increased cell death. Therefore, these studies identified highly cytotoxic mortalin-mimetic peptides that may be used as monotherapy or in combination with complement-activating antibody therapy to target mortalin for precision cancer therapy. Therefore, the reported mortalin-mimetic peptides may be combined as adjuvants with complement-activating antibody therapy to better target breast cancer.

### Antibodies

Antibody internalization is required for the success of many targeted therapeutics, such as immunotoxins, immunoliposomes, antibody–drug conjugates, and for targeted delivery of genes or viral DNA into cells (Nielsen and Marks, 2000). Shitota et al. reported that mortalin is expressed in aggressive and metastatic cancer cells and can bind to unique anti-mortalin antibodies (MotAbs), leading to their internalization into cells (Shiota et al., 2007). Internalizing mortalin Ab (i-mot Ab) can be employed for the internalization of quantum dots (Qdots) (Kaul Z et al., 2007). The Qdot-conjugated MotAb was visible even after multiple divisions and was nontoxic to cells, making it a sensitive tool for long-term molecular imaging. More importantly, owing to its ability to internalize into cancer cells, i-mot Ab has been suggested as a novel vehicle for the targeted delivery of candidate anticancer drugs. The cationic polymer polyethylenimine (PEI) and i-mot Ab complex have been employed for gene delivery, demonstrating enhanced transgene expression in mortalin-positive cells (Shiota et al., 2007). These studies suggest that the i-mot Ab can be a promising target moiety for the vehicle of candidate anticancer drugs. To this end, i-mot Ab was utilized to generate mortalin-targeting nanoparticles loaded with CAPE, which has been previously discussed as a natural inhibitor of the mortalin–p53 interaction (Wang et al., 2020). In this study, the specific interaction between i-mot Ab on the surface of vehicle and mortalin on the surface of cancer cells increased the cellular uptake of CAPE into cancer cells, causing a strong dose-dependent growth arrest and apoptosis of cancer cells and downregulation of proteins involved in cell migration. As a result, CAPE-MotAb revealed significantly enhanced suppression of tumor growth, indicating that these novel CAPE-MotAb nanoparticles may serve as potent anticancer nanomedicines.

### Nucleic Acids

Lu et al. found mortalin–p53 interactions in liver tumors and five hepatocellular carcinoma (HCC) cell lines harboring mutant p53. They showed that the mortalin-p53 interaction is absent in normal liver and immortalized normal hepatocytes. These phenomena indicated that mortalin–p53 interactions is cancer specific, making it a promising target for cancer treatment (Lu et al., 2011a; Lu et al., 2011b). Based on these results, they established mortalin targeting strategies using small hairpin RNA (shRNAs). In HCC, shRNA-mediated mortalin silencing induces mutant p53-mediated tumor-specific apoptosis (Lu et al., 2011a). Based on these results, several studies have used shRNA to develop potent mortalin-targeting strategies. shRNA against mortalin was utilized to enhance chemosensitization of cisplatin into cancer cell lines (Lu et al., 2011b). Furthermore, dual targeting of mortalin (HSC70) and HSP72 inhibits HSP90, which plays a key role in ensuring the correct conformation, stability, and activity of many well-defined oncogenic client proteins, ultimately resulting in the induction of tumor-specific apoptosis (Powers et al., 2008). Furthermore, mortalin was knocked down by specific shRNA-sensitized cells, making it less migratory and more responsive to a variety of chemotherapeutic drugs (Yun et al., 2017). shRNA silencing of mortalin reduced cancer cell stemness by downregulating ABCG2, OCT-4, CD133, ALDH1, CD9, MRP1, and connexin expression levels, leading to a higher ability to form spheroids (Yun et al., 2017). These studies suggest that targeting mortalin-p53 interactions using shRNA may be a promising strategy for cancer therapy.

**FIGURE 1:**
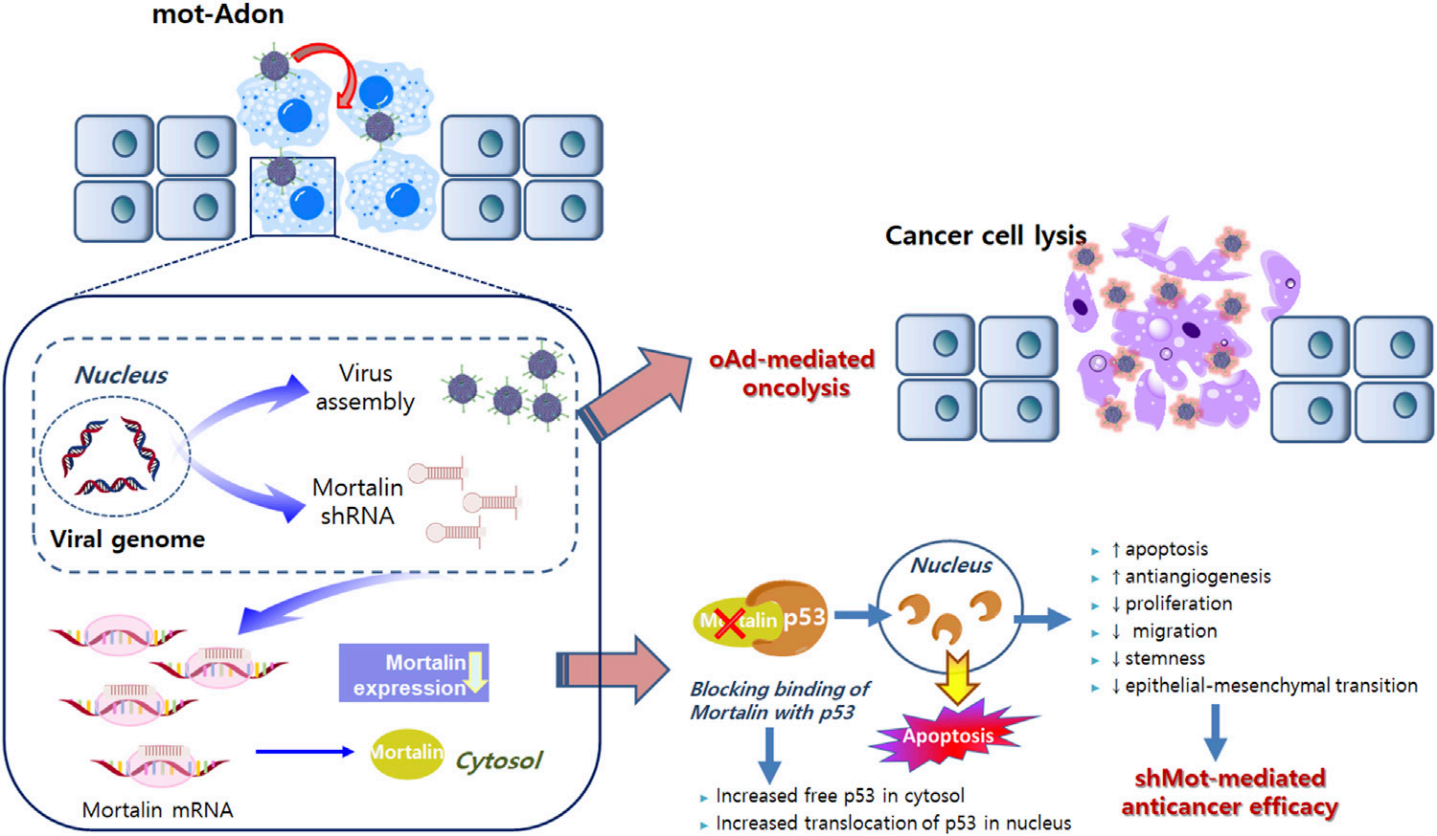
Mortalin-expressing oncolytic adenovirus. Active infection and replication of oncolytic adenovirus in tumor cells results in tumor-specific viral replication and oncolysis. Along with viral replication, the expression of shorthairpin RNA (shRNA) against mortalin is also restricted in cancer cells, resulting in decreased non-specific expression of shRNA and adverse side effects. shRNA against mortalin can reduce the proliferation, migration, stemness, and epithelial–mesenchymal transition, while enhancing the anti-angiogenesis and apoptosis in cancer cells. Collectively, oncolytic adenovirus-mediated oncolytic properties and shRNA against mortalin-mediated anticancer properties can synergistically regulate the anti-tumor effects on cancer cells.

Although the shRNA technique that offers sequence-specific degradation of messenger RNA has been successfully applied to cancer owing to its high specificity and efficacy, the application of small interfering RNA (siRNA) in clinical settings remains challenging owing to its short half-life (Izquierdo, 2005; Kanasty et al., 2013). In these premises, developments of efficient siRNA delivery systems that overcome this limitation have been initiated (Kanasty et al., 2013). One of these is a shRNA-expressing viral vector system, in which vector-mediated expression of shRNA provides prolonged and high levels of RNAi expression in target tissues. Among several viral vectors currently being evaluated in various phases of clinical trials, an oncolytic adenovirus is preferred because of its ability to preferentially replicate in and lyse cancer cells. (Kim et al., 2002; Kim et al., 2003; Kasala et al., 2014). To this end, we previously demonstrated that the delivery of shRNA via the oncolytic adenovirus led to effective silencing of target genes in cancer cells and reduced tumor growth (Yoo et al., 2007; Yoo et al., 2008; Lee et al., 2015). The oncolytic adenovirus, which harbors the E1A and E1B-double mutation and expresses shRNA against mortalin (mot-Adon), was constructed (Yoo et al., 2007). Mot-Adon efficiently downregulated mortalin expression and elicited enhanced cancer cell-killing effect, without affecting the normal cells. More importantly, it demonstrated a potent anti-tumor effect in a mortalin-overexpressing breast tumor model. The anti-tumor effect is caused by enhanced apoptosis, reactivation of p53, and suppression of microvessel formation. Together, these results demonstrate that the oncolytic adenoviral vector combined with shRNA against mortalin can be used as a new platform for tumor therapy (Figure 1).

## Conclusion and Future Perspectives

This review highlights the critical role of mortalin in cancer biology and the significant improvements made in the development of a therapeutic platform to target mortalin. These improvements have greatly enhanced the efficacy of mortalin-targeted cancer therapies. However, each approach still needs to be assessed to verify its safety. To assess whether mortalin is suitable as a drug target in clinical settings, the following criteria should be fulfilled (Deocaris et al., 2013). First, the role of mortalin during cancer progression should be well defined in a tissue- and stage-dependent manner. Second, the therapeutic outcome and safety profiles of targeting mortalin in a therapeutic protocol must be determined. To achieve this, it is crucial to establish a detailed treatment scheme, specifying the number/interval/cycle/route of treatment and combination drugs that maximize the therapeutic efficacy of mortalin targeting. Moreover, the pharmacokinetic properties, biodegradability, biodistribution, and toxicity of mortalin-targeting strategies must be assessed. Finally, favorable surface properties that facilitate therapeutic platform docking with high affinity and specificity should be determined. Collectively, the aforementioned criteria of mortalin-targeting therapeutic platforms that require innovative designs that incorporate the specific strengths of each approach while mitigating their individual drawbacks must be extensively studied to facilitate the use of such mortalin-targeting therapeutic platforms in future clinical trials.

## References

[B1] Abdullah A. Sane S. Branick K. A. Freeling J. L. Wang H. Zhang D. (2015). A Plant Alkaloid, Veratridine, Potentiates Cancer Chemosensitivity by UBXN2A-Dependent Inhibition of an Oncoprotein, Mortalin-2. Oncotarget 6 (27), 23561–23581. 10.18632/oncotarget.4452 PubMed Abstract | 10.18632/oncotarget.4452 | Google Scholar 26188124 PMC4695137

[B2] Adams J. D. Jr. Yang J. Mishra L. C. Singh B. B. (2002). Effects of Ashwagandha in a Rat Model of Stroke. Altern. Ther. Health Med. 8 (5), 18–19. Google Scholar 12233797

[B3] Bhargava P. Grover A. Nigam N. Kaul A. Doi M. Ishida Y. (2018). Anticancer Activity of the Supercritical Extract of Brazilian Green Propolis and its Active Component, Artepillin C: Bioinformatics and Experimental Analyses of its Mechanisms of Action. Int. J. Oncol. 52 (3), 925–932. 10.3892/ijo.2018.4249 PubMed Abstract | 10.3892/ijo.2018.4249 | Google Scholar 29393408

[B4] Bhargava P. Malik V. Liu Y. Ryu J. Kaul S. C. Sundar D. (2019). Molecular Insights into Withaferin-A-Induced Senescence: Bioinformatics and Experimental Evidence to the Role of NFκB and CARF. J. Gerontol. A Biol. Sci. Med. Sci. 74 (2), 183–191. 10.1093/gerona/gly107 PubMed Abstract | 10.1093/gerona/gly107 | Google Scholar 29718136

[B5] Bykov V. J. N. Zache N. Stridh H. Westman J. Bergman J. Selivanova G. (2005). PRIMA-1MET Synergizes with Cisplatin to Induce Tumor Cell Apoptosis. Oncogene 24 (21), 3484–3491. 10.1038/sj.onc.1208419 PubMed Abstract | 10.1038/sj.onc.1208419 | Google Scholar 15735745

[B6] Deocaris C. Lu W.-J. Kaul S. Wadhwa R. (2013). Druggability of Mortalin for Cancer and Neuro-Degenerative Disorders. Curr. Pharm. Des. 19 (3), 418–429. 10.2174/138161213804143680 PubMed Abstract | 10.2174/138161213804143680 | Google Scholar 22920904

[B7] Denu R. A. Zasadil L. M. Kanugh C. Laffin J. Weaver B. A. Burkard M. E. (2016). Centrosome Amplification Induces High Grade Features and Is Prognostic of Worse Outcomes in Breast Cancer. BMC Cancer 16, 47. 10.1186/s12885-016-2083-x PubMed Abstract | 10.1186/s12885-016-2083-x | Google Scholar 26832928 PMC4734858

[B8] Elwakeel A. Sari A. N. Dhanjal J. K. Meidinna H. N. Sundar D. Kaul S. C. (2021). Mutant p53L194F Harboring Luminal-A Breast Cancer Cells Are Refractory to Apoptosis and Cell Cycle Arrest in Response to MortaparibPlus, a Multimodal Small Molecule InhibitorHarboring Luminal-A Breast Cancer Cells Are Refractory to Apoptosis and Cell Cycle Arrest in Response to Mortaparib(Plus), a Multimodal Small Molecule Inhibitor. Cancers (Basel). 13 (12), 3043. 10.3390/cancers13123043 PubMed Abstract | 10.3390/cancers13123043 | Google Scholar 34207240 PMC8234533

[B9] Gao R. Shah N. Lee J.-S. Katiyar S. P. Li L. Oh E. (2014). Withanone-Rich Combination of Ashwagandha Withanolides Restricts Metastasis and Angiogenesis Through hnRNP-K. Mol. Cancer Ther. 13 (12), 2930–2940. 10.1158/1535-7163.mct-14-0324 PubMed Abstract | 10.1158/1535-7163.mct-14-0324 | Google Scholar 25236891

[B10] Garg S. Afzal S. Elwakeel A. Sharma D. Radhakrishnan N. Dhanjal J. K. (2019). Marine Carotenoid Fucoxanthin Possesses Anti-Metastasis Activity: Molecular Evidence. Mar. Drugs 17 (6), 338. 10.3390/md17060338 PubMed Abstract | 10.3390/md17060338 | Google Scholar PMC6627158 31195739

[B11] Guruswamy S. Lightfoot S. Gold M. A. Hassan R. Berlin K. D. Ivey R. T. (2001). Effects of Retinoids on Cancerous Phenotype and Apoptosis in Organotypic Cultures of Ovarian Carcinoma. J. Natl. Cancer Inst. 93 (7), 516–525. 10.1093/jnci/93.7.516 PubMed Abstract | 10.1093/jnci/93.7.516 | Google Scholar 11287445

[B12] Huang M.-B. Gonzalez R. R. Lillard J. Bond V. C. (2017). Secretion Modification Region-Derived Peptide Blocks Exosome Release and Mediates Cell Cycle Arrest in Breast Cancer Cells. Oncotarget 8 (7), 11302–11315. 10.18632/oncotarget.14513 PubMed Abstract | 10.18632/oncotarget.14513 | Google Scholar 28076321 PMC5355266

[B13] Ichihara M. Murakumo Y. Takahashi M. (2004). RET and Neuroendocrine Tumors. Cancer Lett. 204 (2), 197–211. 10.1016/s0304-3835(03)00456-7 PubMed Abstract | 10.1016/s0304-3835(03)00456-7 | Google Scholar 15013219

[B14] Iosefson O. Azem A. (2010). Reconstitution of the Mitochondrial Hsp70 (Mortalin)-p53 Interaction Using Purified Proteins--Identification of Additional Interacting Regions. FEBS Lett. 584 (6), 1080–1084. 10.1016/j.febslet.2010.02.019 PubMed Abstract | 10.1016/j.febslet.2010.02.019 | Google Scholar 20153329

[B15] Ishida Y. Gao R. Shah N. Bhargava P. Furune T. Kaul S. C. (2018). Anticancer Activity in Honeybee Propolis: Functional Insights to the Role of Caffeic Acid Phenethyl Ester and its Complex With γ-Cyclodextrin. Integr. Cancer Ther. 17 (3), 867–873. 10.1177/1534735417753545 PubMed Abstract | 10.1177/1534735417753545 | Google Scholar 29390900 PMC6142091

[B16] Izquierdo M. (2005). Short Interfering RNAs as a Tool for Cancer Gene Therapy. Cancer Gene Ther. 12 (3), 217–227. 10.1038/sj.cgt.7700791 PubMed Abstract | 10.1038/sj.cgt.7700791 | Google Scholar 15550938

[B17] Jubran R. Saar-Ray M. Wawruszak A. Ziporen L. Donin N. Bairey O. (2020). Mortalin Peptides Exert Antitumor Activities and Act as Adjuvants to Antibody-Mediated Complement-dependent Cytotoxicity. Int. J. Oncol. 57 (4), 1013–1026. 10.3892/ijo.2020.5101 PubMed Abstract | 10.3892/ijo.2020.5101 | Google Scholar 32700755

[B18] Kanasty R. Dorkin J. R. Vegas A. Anderson D. (2013). Delivery Materials for siRNA Therapeutics. Nat. Mater 12 (11), 967–977. 10.1038/nmat3765 PubMed Abstract | 10.1038/nmat3765 | Google Scholar 24150415

[B19] Kasala D. Choi J.-W. Kim S. W. Yun C.-O. (2014). Utilizing Adenovirus Vectors for Gene Delivery in Cancer. Expert Opin. Drug Deliv. 11 (3), 379–392. 10.1517/17425247.2014.874414 PubMed Abstract | 10.1517/17425247.2014.874414 | Google Scholar 24392755

[B20] Kaul S Deocaris C. C. Wadhwa R. (2007). Three Faces of Mortalin: a Housekeeper, Guardian and Killer. Exp. Gerontol. 42 (4), 263–274. 10.1016/j.exger.2006.10.020 PubMed Abstract | 10.1016/j.exger.2006.10.020 | Google Scholar 17188442

[B21] Kaul Z. Yaguchi T. Harada J. I. Ikeda Y. Hirano T. Chiura H. X. (2007). An Antibody-Conjugated Internalizing Quantum Dot Suitable for Long-Term Live Imaging of Cells. Biochem. Cell Biol. 85 (1), 133–140. 10.1139/o06-205 PubMed Abstract | 10.1139/o06-205 | Google Scholar 17464353

[B22] Kaul S. C. Duncan E. L. Englezou A. Takano S. Reddel R. R. Mitsui Y. (1998). Malignant Transformation of NIH3T3 Cells by Overexpression of Mot-2 Protei. Oncogene 17 (7), 907–911. 10.1038/sj.onc.1202017 PubMed Abstract | 10.1038/sj.onc.1202017 | Google Scholar 9780007

[B23] Kaul S. C. Yaguchi T. Taira K. Reddel R. R. Wadhwa R. (2003). Overexpressed Mortalin (mot-2)/mthsp70/GRP75 and hTERT Cooperate to Extend the *In Vitro* Lifespan of Human Fibroblasts. Exp. Cell Res. 286 (1), 96–101. 10.1016/s0014-4827(03)00101-0 PubMed Abstract | 10.1016/s0014-4827(03)00101-0 | Google Scholar 12729798

[B24] Kim E. Kim J.-H. Shin H.-Y. Lee H. Yang J. M. Kim J. (2003). Ad-mTERT-Δ19, a Conditional Replication-Competent Adenovirus Driven by the Human Telomerase Promoter, Selectively Replicates in and Elicits Cytopathic Effect in a Cancer Cell-specific Manner. Hum. Gene Ther. 14 (15), 1415–1428. 10.1089/104303403769211637 PubMed Abstract | 10.1089/104303403769211637 | Google Scholar 14577922

[B25] Kim J. Cho J. Y. Kim J.-H. Jung K. C. Yun C.-O. (2002). Evaluation of E1B Gene-Attenuated Replicating Adenoviruses for Cancer Gene Therapy. Cancer Gene Ther. 9 (9), 725–736. 10.1038/sj.cgt.7700494 PubMed Abstract | 10.1038/sj.cgt.7700494 | Google Scholar 12189522

[B26] Kim S.-H. Hahm E.-R. Arlotti J. A. Samanta S. K. Moura M. B. Thorne S. H. (2016). Withaferin A Inhibits *In Vivo* Growth of Breast Cancer Cells Accelerated by Notch2 Knockdown. Breast Cancer Res. Treat. 157 (1), 41–54. 10.1007/s10549-016-3795-y PubMed Abstract | 10.1007/s10549-016-3795-y | Google Scholar 27097807 PMC4867258

[B27] Koduru S. Kumar R. Srinivasan S. Evers M. B. Damodaran C. (2010). Notch-1 Inhibition by Withaferin-A: a Therapeutic Target against Colon Carcinogenesis. Mol. Cancer Ther. 9 (1), 202–210. 10.1158/1535-7163.mct-09-0771 PubMed Abstract | 10.1158/1535-7163.mct-09-0771 | Google Scholar 20053782 PMC3041017

[B28] Koya K. Li Y. Wang H. Ukai T. Tatsuta N. Kawakami M. (1996). MKT-077, a Novel Rhodacyanine Dye in Clinical Trials, Exhibits Anticarcinoma Activity in Preclinical Studies Based on Selective Mitochondrial Accumulation. Cancer Res. 56 (3), 538–543. PubMed Abstract | Google Scholar 8564968

[B29] Kulkarni S. K. Dhir A. (2008). Withania Somnifera: an Indian Ginseng. Prog. Neuro-Psychopharmacology Biol. Psychiatry 32 (5), 1093–1105. 10.1016/j.pnpbp.2007.09.011 10.1016/j.pnpbp.2007.09.011 | Google Scholar 17959291

[B30] Lee J.-S. Oh E. Yoo J. Y. Choi K. S. Yoon M. J. Yun C.-O. (2015). Adenovirus Expressing Dual C-Met-specific shRNA Exhibits Potent Antitumor Effect Through Autophagic Cell Death Accompanied by Senescence-like Phenotypes in Glioblastoma Cells. Oncotarget 6 (6), 4051–4065. 10.18632/oncotarget.3018 PubMed Abstract | 10.18632/oncotarget.3018 | Google Scholar 25726528 PMC4414172

[B31] Li Y. Xu Y. Ling M. Yang Y. Wang S. Li Z. (2010). mot-2-Mediated Cross Talk between Nuclear Factor-Κb and P53 Is Involved in Arsenite-Induced Tumorigenesis of Human Embryo Lung Fibroblast Cells. Environ. Health Perspect. 118 (7), 936–942. 10.1289/ehp.0901677 PubMed Abstract | 10.1289/ehp.0901677 | Google Scholar 20199942 PMC2920912

[B32] Liu T. Masamha C. P. Chengedza S. Berlin K. D. Lightfoot S. He F. (2009). Development of Flexible-Heteroarotinoids for Kidney Cancer. Mol. Cancer Ther. 8 (5), 1227–1238. 10.1158/1535-7163.mct-08-1069 PubMed Abstract | 10.1158/1535-7163.mct-08-1069 | Google Scholar 19417155 PMC2888972

[B33] Lu W.-J. Lee N. P. Kaul S. C. Lan F. Poon R. T. P. Wadhwa R. (2011a). Induction of Mutant P53-dependent Apoptosis in Human Hepatocellular Carcinoma by Targeting Stress Protein Mortalin. Int. J. Cancer 129 (8), 1806–1814. 10.1002/ijc.25857 PubMed Abstract | 10.1002/ijc.25857 | Google Scholar 21165951

[B34] Lu W.-J. Lee N. P. Kaul S. C. Lan F. Poon R. T. P. Wadhwa R. (2011b). Mortalin-p53 Interaction in Cancer Cells Is Stress Dependent and Constitutes a Selective Target for Cancer Therapy. Cell Death Differ. 18 (6), 1046–1056. 10.1038/cdd.2010.177 PubMed Abstract | 10.1038/cdd.2010.177 | Google Scholar 21233847 PMC3131943

[B35] Ma Z. Izumi H. Kanai M. Kabuyama Y. Ahn N. G. Fukasawa K. (2006). Mortalin Controls Centrosome Duplication via Modulating Centrosomal Localization of P53. Oncogene 25 (39), 5377–5390. 10.1038/sj.onc.1209543 PubMed Abstract | 10.1038/sj.onc.1209543 | Google Scholar 16619038

[B36] Malik F. Singh J. Khajuria A. Suri K. A. Satti N. K. Singh S. (2007). A Standardized Root Extract of Withania Somnifera and its Major Constituent Withanolide-A Elicit Humoral and Cell-Mediated Immune Responses by up Regulation of Th1-Dominant Polarization in BALB/c Mice. Life Sci. 80 (16), 1525–1538. 10.1016/j.lfs.2007.01.029 PubMed Abstract | 10.1016/j.lfs.2007.01.029 | Google Scholar 17336338

[B37] Mantovani F. Collavin L. Del Sal G. (2019). Mutant P53 as a Guardian of the Cancer Cell. Cell Death Differ. 26 (2), 199–212. 10.1038/s41418-018-0246-9 PubMed Abstract | 10.1038/s41418-018-0246-9 | Google Scholar 30538286 PMC6329812

[B38] Masamha C. P. Benbrook D. M. (2009). Cyclin D1 Degradation Is Sufficient to Induce G1 Cell Cycle Arrest Despite Constitutive Expression of Cyclin E2 in Ovarian Cancer Cells. Cancer Res. 69 (16), 6565–6572. 10.1158/0008-5472.can-09-0913 PubMed Abstract | 10.1158/0008-5472.can-09-0913 | Google Scholar 19638577

[B39] Miyata Y. Li X. Lee H.-F. Jinwal U. K. Srinivasan S. R. Seguin S. P. (2013). Synthesis and Initial Evaluation of YM-08, a Blood-Brain Barrier Permeable Derivative of the Heat Shock Protein 70 (Hsp70) Inhibitor MKT-077, Which Reduces Tau Levels. ACS Chem. Neurosci. 4 (6), 930–939. 10.1021/cn300210g PubMed Abstract | 10.1021/cn300210g | Google Scholar 23472668 PMC3689201

[B40] Mylonis I. Kourti M. Samiotaki M. Panayotou G. Simos G. (2017). Mortalin-mediated and ERK-Controlled Targeting of HIF-1α to Mitochondria Confers Resistance to Apoptosis under Hypoxia. J. Cell Sci. 130 (2), 466–479. 10.1242/jcs.195339 PubMed Abstract | 10.1242/jcs.195339 | Google Scholar 27909249

[B41] Na Y. Kaul S. C. Ryu J. Lee J.-S. Ahn H. M. Kaul Z. (2016). Stress Chaperone Mortalin Contributes to Epithelial-To-Mesenchymal Transition and Cancer Metastasis. Cancer Res. 76 (9), 2754–2765. 10.1158/0008-5472.can-15-2704 PubMed Abstract | 10.1158/0008-5472.can-15-2704 | Google Scholar 26960973

[B42] Nielsen U. B. Marks J. D. (2000). Internalizing Antibodies and Targeted Cancer Therapy: Direct Selection from Phage Display Libraries. Pharm. Sci. Technol. Today 3 (8), 282–291. 10.1016/s1461-5347(00)00280-7 PubMed Abstract | 10.1016/s1461-5347(00)00280-7 | Google Scholar 10916148

[B43] Nigam N. Grover A. Goyal S. Katiyar S. P. Bhargava P. Wang P.-C. (2015). Targeting Mortalin by Embelin Causes Activation of Tumor Suppressor P53 and Deactivation of Metastatic Signaling in Human Breast Cancer Cells. Plos One 10 (9), e0138192. 10.1371/journal.pone.0138192 PubMed Abstract | 10.1371/journal.pone.0138192 | Google Scholar 26376435 PMC4574062

[B44] Oh J. H. Lee T.-J. Kim S. H. Choi Y. H. Lee S. H. Lee J. M. (2008). Induction of Apoptosis by Withaferin A in Human Leukemia U937 Cells through Down-Regulation of Akt Phosphorylation. Apoptosis 13 (12), 1494–1504. 10.1007/s10495-008-0273-y PubMed Abstract | 10.1007/s10495-008-0273-y | Google Scholar 19002588

[B45] Powers M. V. Clarke P. A. Workman P. (2008). Dual Targeting of HSC70 and HSP72 Inhibits HSP90 Function and Induces Tumor-specific Apoptosis. Cancer Cell 14 (3), 250–262. 10.1016/j.ccr.2008.08.002 PubMed Abstract | 10.1016/j.ccr.2008.08.002 | Google Scholar 18772114

[B46] Putri J. F. Bhargava P. Dhanjal J. K. Yaguchi T. Sundar D. Kaul S. C. (2019). Mortaparib, a Novel Dual Inhibitor of Mortalin and PARP1, Is a Potential Drug Candidate for Ovarian and Cervical Cancers. J. Exp. Clin. Cancer Res. 38 (1), 499. 10.1186/s13046-019-1500-9 PubMed Abstract | 10.1186/s13046-019-1500-9 | Google Scholar 31856867 PMC6923857

[B47] Ramraj S. K. Elayapillai S. P. Pelikan R. C. Zhao Y. D. Isingizwe Z. R. Kennedy A. L. (2020). Novel Ovarian Cancer Maintenance Therapy Targeted at Mortalin and Mutant P53. Int. J. Cancer 147 (4), 1086–1097. 10.1002/ijc.32830 PubMed Abstract | 10.1002/ijc.32830 | Google Scholar 31845320 PMC7297651

[B48] Ran Q. Wadhwa R. Kawai R. Kaul S. C. Sifers R. N. Bick R. J. (2000). Extramitochondrial Localization of mortalin/mthsp70/PBP74/GRP75. Biochem. Biophysical Res. Commun. 275 (1), 174–179. 10.1006/bbrc.2000.3237 PubMed Abstract | 10.1006/bbrc.2000.3237 | Google Scholar 10944461

[B49] Rozenberg P. Kocsis J. Saar M. Prohászka Z. Füst G. Fishelson Z. (2013). Elevated Levels of Mitochondrial Mortalin and Cytosolic HSP70 in Blood as Risk Factors in Patients with Colorectal Cancer. Int. J. Cancer 133 (2), 514–518. 10.1002/ijc.28029 PubMed Abstract | 10.1002/ijc.28029 | Google Scholar 23319326

[B50] Ryu J. Kaul Z. Yoon A.-R. Liu Y. Yaguchi T. Na Y. (2014). Identification and Functional Characterization of Nuclear Mortalin in Human Carcinogenesis. J. Biol. Chem. 289 (36), 24832–24844. 10.1074/jbc.m114.565929 PubMed Abstract | 10.1074/jbc.m114.565929 | Google Scholar 25012652 PMC4155653

[B51] Sari A. N. Bhargava P. Dhanjal J. K. Putri J. F. Radhakrishnan N. Shefrin S. (2020). Combination of Withaferin-A and CAPE Provides Superior Anticancer Potency: Bioinformatics and Experimental Evidence to Their Molecular Targets and Mechanism of Action. Cancers (Basel) 12 (5), 1160. 10.3390/cancers12051160 PubMed Abstract | 10.3390/cancers12051160 | Google Scholar PMC7281427 32380701

[B52] Sen N. Banerjee B. Das B. B. Ganguly A. Sen T. Pramanik S. (2007). Apoptosis Is Induced in Leishmanial Cells by a Novel Protein Kinase Inhibitor Withaferin A and Is Facilitated by Apoptotic Topoisomerase I-DNA Complex. Cell Death Differ. 14 (2), 358–367. 10.1038/sj.cdd.4402002 PubMed Abstract | 10.1038/sj.cdd.4402002 | Google Scholar 16841091

[B53] Shelton M. N. Huang M.-B. Ali S. A. Powell M. D. Bond V. C. (2012). Secretion Modification Region-Derived Peptide Disrupts HIV-1 Nef's Interaction with Mortalin and Blocks Virus and Nef Exosome Release. J. Virol. 86 (1), 406–419. 10.1128/jvi.05720-11 PubMed Abstract | 10.1128/jvi.05720-11 | Google Scholar 22013042 PMC3255900

[B54] Shiota M. Ikeda Y. Kaul Z. Itadani J. Kaul S. C. Wadhwa R. (2007). Internalizing Antibody-Based Targeted Gene Delivery for Human Cancer Cells. Hum. Gene Ther. 18 (11), 1153–1160. 10.1089/hum.2007.087 PubMed Abstract | 10.1089/hum.2007.087 | Google Scholar 17937579

[B55] Srinivasan S. Ranga R. S. Burikhanov R. Han S.-S. Chendil D. (2007). Par-4-dependent Apoptosis by the Dietary Compound Withaferin A in Prostate Cancer Cells. Cancer Res. 67 (1), 246–253. 10.1158/0008-5472.can-06-2430 PubMed Abstract | 10.1158/0008-5472.can-06-2430 | Google Scholar 17185378

[B56] Tikoo A. Cutler H. Lo S. H. Chen L. B. Maruta H. (1999). Treatment of Ras-Induced Cancers by the F-Actin Cappers Tensin and Chaetoglobosin K, in Combination with the Caspase-1 Inhibitor N1445. Cancer J. Sci. Am. 5 (5), 293–300. PubMed Abstract | Google Scholar 10526670

[B57] Tikoo A. Shakri R. Connolly L. Hirokawa Y. Shishido T. Bowers B. (2000). Treatment of Ras-Induced Cancers by the F-Actin-Bundling Drug MKT-077. Cancer J. 6 (3), 162–168. PubMed Abstract | Google Scholar 10882332

[B58] Vaishnavi K. Saxena N. Shah N. Singh R. Manjunath K. Uthayakumar M. (2012). Differential Activities of the Two Closely Related Withanolides, Withaferin A and Withanone: Bioinformatics and Experimental Evidences. Plos One 7 (9), e44419. 10.1371/journal.pone.0044419 PubMed Abstract | 10.1371/journal.pone.0044419 | Google Scholar 22973447 PMC3433425

[B59] Wadhwa R. Sugihara T. Yoshida A. Nomura H. Reddel R. R. Simpson R. (2000). Selective Toxicity of MKT-077 to Cancer Cells Is Mediated by its Binding to the Hsp70 Family Protein Mot-2 and Reactivation of P53 Function. Cancer Res. 60 (24), 6818–6821. PubMed Abstract | Google Scholar 11156371

[B60] Wadhwa R. Kaul S. C. Ikawa Y. Sugimoto Y. (1993a). Identification of a Novel Member of Mouse Hsp70 Family. Its Association with Cellular Mortal Phenotype. J. Biol. Chem. 268 (9), 6615–6621. 10.1016/s0021-9258(18)53295-6 10.1016/s0021-9258(18)53295-6 | Google Scholar 8454632

[B61] Wadhwa R. Kaul S. C. Sugimoto Y. Mitsui Y. (1993b). Induction of Cellular Senescence by Transfection of Cytosolic Mortalin cDNA in NIH 3T3 Cells. J. Biol. Chem. 268 (30), 22239–22242. 10.1016/s0021-9258(18)41515-3 PubMed Abstract | 10.1016/s0021-9258(18)41515-3 | Google Scholar 7693662

[B62] Wadhwa R. Nigam N. Bhargava P. Dhanjal J. K. Goyal S. Grover A. (2016). Molecular Characterization and Enhancement of Anticancer Activity of Caffeic Acid Phenethyl Ester by γ Cyclodextrin. J. Cancer 7 (13), 1755–1771. 10.7150/jca.15170 PubMed Abstract | 10.7150/jca.15170 | Google Scholar 27698914 PMC5039358

[B63] Wadhwa R. Taira K. Kaul S. C. (2002). An Hsp70 Family Chaperone, mortalin/mthsp70/PBP74/Grp75: what, when, and where? Cell Stress Chaper 7 (3), 309–316. 10.1379/1466-1268(2002)007<0309:ahfcmm>2.0.co;2 PubMed Abstract | 10.1379/1466-1268(2002)007<0309:ahfcmm>2.0.co;2 | Google Scholar PMC514830 12482206

[B64] Wadhwa R. Takano S. Kaur K. Deocaris C. C. Pereira-Smith O. M. Reddel R. R. (2006). Upregulation of mortalin/mthsp70/Grp75 Contributes to Human Carcinogenesis. Int. J. Cancer 118 (12), 2973–2980. 10.1002/ijc.21773 PubMed Abstract | 10.1002/ijc.21773 | Google Scholar 16425258

[B65] Wadhwa R. Takano S. Robert M. Yoshida A. Nomura H. Reddel R. R. (1998). Inactivation of Tumor Suppressor P53 by Mot-2, a Hsp70 Family Member. J. Biol. Chem. 273 (45), 29586–29591. 10.1074/jbc.273.45.29586 PubMed Abstract | 10.1074/jbc.273.45.29586 | Google Scholar 9792667

[B66] Wang J. Bhargava P. Yu Y. Sari A. N. Zhang H. Ishii N. (2020). Novel Caffeic Acid Phenethyl Ester-Mortalin Antibody Nanoparticles Offer Enhanced Selective Cytotoxicity to Cancer Cells. Cancers (Basel) 12 (9), 2370. 10.3390/cancers12092370 PubMed Abstract | 10.3390/cancers12092370 | Google Scholar PMC7564736 32825706

[B67] Wang L. Zeng Y. Liu Y. Hu X. Li S. Wang Y. (2014). Fucoxanthin Induces Growth Arrest and Apoptosis in Human Bladder Cancer T24 Cells by Up-Regulation of P21 and Down-Regulation of Mortalin. Acta Biochim. Biophys. Sin. (Shanghai) 46 (10), 877–884. 10.1093/abbs/gmu080 PubMed Abstract | 10.1093/abbs/gmu080 | Google Scholar 25187415

[B68] Widodo N. Priyandoko D. Shah N. Wadhwa R. Kaul S. C. (2010). Selective Killing of Cancer Cells by Ashwagandha Leaf Extract and its Component Withanone Involves ROS Signaling. Plos One 5 (10), e13536. 10.1371/journal.pone.0013536 PubMed Abstract | 10.1371/journal.pone.0013536 | Google Scholar 20975835 PMC2958829

[B69] Widodo N. Takagi Y. Shrestha B. G. Ishii T. Kaul S. C. Wadhwa R. (2008). Selective Killing of Cancer Cells by Leaf Extract of Ashwagandha: Components, Activity and Pathway Analyses. Cancer Lett. 262 (1), 37–47. 10.1016/j.canlet.2007.11.037 PubMed Abstract | 10.1016/j.canlet.2007.11.037 | Google Scholar 18191020

[B70] Wu P.-K. Hong S.-K. Veeranki S. Karkhanis M. Starenki D. Plaza J. A. (2013). A mortalin/HSPA9-Mediated Switch in Tumor-Suppressive Signaling of Raf/MEK/extracellular Signal-Regulated Kinase. Mol. Cell Biol. 33 (20), 4051–4067. 10.1128/mcb.00021-13 PubMed Abstract | 10.1128/mcb.00021-13 | Google Scholar 23959801 PMC3811686

[B71] Yang H. Shi G. Dou Q. P. (2007). The Tumor Proteasome Is a Primary Target for the Natural Anticancer Compound Withaferin A Isolated from "Indian Winter Cherry". Mol. Pharmacol. 71 (2), 426–437. 10.1124/mol.106.030015 PubMed Abstract | 10.1124/mol.106.030015 | Google Scholar 17093135

[B72] Yang H. Wang Y. Cheryan V. T. Wu W. Cui C. Q. Polin L. A. (2012). Withaferin A Inhibits the Proteasome Activity in Mesothelioma *In Vitro* and *In Vivo* . Plos One 7 (8), e41214. 10.1371/journal.pone.0041214 PubMed Abstract | 10.1371/journal.pone.0041214 | Google Scholar 22912669 PMC3422308

[B73] Yang L. Guo W. Zhang Q. Li H. Liu X. Yang Y. (2011). Crosstalk between Raf/MEK/ERK and PI3K/AKT in Suppression of Bax Conformational Change by Grp75 under Glucose Deprivation Conditions. J. Mol. Biol. 414 (5), 654–666. 10.1016/j.jmb.2011.09.009 PubMed Abstract | 10.1016/j.jmb.2011.09.009 | Google Scholar 21964438

[B74] Yokoyama K. Fukumoto K. Murakami T. Harada S. Hosono R. Wadhwa R. (2002). Extended Longevity of *Caenorhabditis elegans* by Knocking in Extra Copies of hsp70F, a Homolog of Mot-2 (mortalin)/mthsp70/Grp75. FEBS Lett. 516 (1-3), 53–57. 10.1016/s0014-5793(02)02470-5 PubMed Abstract | 10.1016/s0014-5793(02)02470-5 | Google Scholar 11959102

[B75] Yoo J. Y. Kim J.-H. Kim J. Huang J.-H. Zhang S. N. Kang Y.-A. (2008). Short Hairpin RNA-Expressing Oncolytic Adenovirus-Mediated Inhibition of IL-8: Effects on Antiangiogenesis and Tumor Growth Inhibition. Gene Ther. 15 (9), 635–651. 10.1038/gt.2008.3 PubMed Abstract | 10.1038/gt.2008.3 | Google Scholar 18273054

[B76] Yoo J. Y. Kim J.-H. Kwon Y.-G. Kim E.-C. Kim N. K. Choi H. J. (2007). VEGF-specific Short Hairpin RNA-Expressing Oncolytic Adenovirus Elicits Potent Inhibition of Angiogenesis and Tumor Growth. Mol. Ther. 15 (2), 295–302. 10.1038/sj.mt.6300023 PubMed Abstract | 10.1038/sj.mt.6300023 | Google Scholar 17235307

[B77] Yun C.-O. Bhargava P. Na Y. Lee J.-S. Ryu J. Kaul S. C. (2017). Relevance of Mortalin to Cancer Cell Stemness and Cancer Therapy. Sci. Rep. 7, 42016. 10.1038/srep42016 PubMed Abstract | 10.1038/srep42016 | Google Scholar 28165047 PMC5292728

