# Supplementary material for: Why is Mortalin a Potential Therapeutic Target for Cancer?
Source: Front Cell Dev Biol. 2022 Jun 29;10:914540. doi: 10.3389/fcell.2022.914540 (PMC9290191; doi:10.3389/fcell.2022.914540)
Supplement: Supplementary file 1 [file Table1.PDF]

Table 1.

Therapeutic platform	Agent	Targeting effects	Reference
Natural compounds	Withaferin A (Wi-A; withanolides from Ashwagandha)	- Induction of apoptosis<br>- Inhibition of Notch-1 signaling<br>- Downregulation of serine-threonine kinase (Akt)/nuclear factor (NF)-kB/B-cell lymphoma (Bcl)-2 pathways<br>- Strong cytotoxicity to both cancer and normal cells<br>- Potent anti-migratory, anti-invasive, and anti-angiogenic activities	[27-33]
	Withanone (Wi-N; withanolides from Ashwagandha)	- Interaction with p53, hmot-2, p21WAF1, and nuclear factor-erythroid 2-related factor 2 (Nrf2) (i-Extract)<br>- Increased selective killing of cancer cells	[33, 37-40]
	CAPE (Ingredient of New Zealand honeybee propolis)	- Disruption of the mortalin–p53 complex<br>- Increased translocation and activation of p53 in the nucleus<br>- Enhancement of anti-tumor and anti-metastasis (γCD-complexed CAPE)	[41, 42, 44]
	Artepillin C (Ingredient of Brazilian green propolis)	- Abrogation of mortalin–p53 complexes, activation of p53<br>- Increased growth arrest of cancer cells<br>- Increased cytotoxicity *in vitro* (green propolis-supercritical extract (GPSE) -conjugated with γCD)<br>- Potent anticancer activity (GPSE-conjugated with γCD)	[45]
	Veratridine (VTD) (Alkaloid derived from Liliaceae plants)	- Increased transactivation of cytoplasmic UBX domain protein 2A (UBXN2A)<br>- Inhibition of motalin<br>- Increased tumor-specific toxicity<br>- Induction of cancer chemosensitivity	[23]
	Embelin (natural quinone from *Embelia ribes*)	- Induction of cancer chemosensitivity<br>- Increased nuclear translocation of p53<br>- Abolished activity of the mortalin–p53 complex<br>- Downregulation of growth factor expression and metastatic signaling pathway<br>- Inhibition of cancer cell growth	[46]
	Fucoxanthin (found in marine organisms)	- Suppression of the transcriptional activity of mortalin<br>- Activation of p53 function in cancer cells<br>- Decreased cancer cell proliferation and survival<br>- Cancer cell-specific killing	[47]

		- Decreased migration and invasion of cancer cells	
Chemical compounds	MKT-077 ($C_{21}H_{22}ClN_3OS_2$)	- Elimination of mortalin–p53 interactions (no alteration of mortalin expression)<br>- Binding to mot-2 and abrogation of its interaction with p53<br>- Enhancement of anti-tumor activity<br>- Overcoming the limited efficacy of photodynamic therapy<br>- Induction of apoptosis or necrosis<br>- Decreased drug resistance of cancer cells	[48-52]
	Mortaparib	- Abrogation of cancer cell-specific mortalin–p53 interactions<br>- Induction of growth arrest/apoptosis signaling<br>- Inhibition of cancer cell migration, metastasis, and angiogenesis *in vitro*<br>- Potent anti-tumor and anti-metastatic effects<br>- A first dual inhibitor of mortalin and poly(ADP-ribose) polymerase 1 (PARP-1)	[53]
	Mortaparib^{Plus}	- Prevention of mortalin–p53 interaction<br>- Induction of growth arrest and apoptosis<br>- Induction of cancer cell death via multiple mechanisms	[54]
	SHetA2	- Disruption of mortalin–p53 complexes in ovarian cancer cells<br>- Induction of apoptosis in cancer cells<br>- Tumor growth inhibition in orthotopic ovarian tumor model (combination with PRIMA-1MET)	[55-59]
Peptide	SMR of Nef	- Disruption of the interaction of Nef with mortalin<br>- Antagonization of the functions of mortalin (SMR-CLU)<br>- Decrease in breast cancer cell metastasis (SMT-CLU)<br>- Inhibition of growth (PEG-SMRwt-CLU)<br>- Cell cycle arrest at G_2/M phase (PEG-SMRwt-CLU)<br>- Increased sensitivity to cisplatin and paclitaxel (SMRwt)	[60, 61]
	Mot-P2 or Mot-P7	- Antibody-mediated and complement-dependent cell killing<br>- Increased plasma membrane perforation and mitochondrial inner membrane depolarization<br>- Decrease in ATP levels<br>- Increased cell death on combination with rituximab	[62]
Antibody	i-mot Ab	- Enhanced transgene expression in mortalin-positive cells (i-mot Ab/PEI complex)<br>- Increased cellular uptake of CAPE (CAPE-MotAb)<br>- Stronger growth arrest/apoptosis (CAPE-MotAb)<br>- Downregulation of the expression levels of proteins involved in cell migration (CAPE-MotAb)	[64, 66]

This is a provisional file, not the final typeset article

		- Significant suppression of tumor growth (CAPE-MotAb)	
Nucleic acid	shRNA against motalin	- Induction of mutant p53-mediated tumor-specific apoptosis<br>- Increased responsiveness of cancer cells to chemotherapeutic drugs<br>- Reduction in cancer cell stemness<br>- Downregulation of the expression levels of ATP binding cassette subfamily G member 2 (ABCG2), POU class 5 homeobox 1 (POU5F1/OCT-4), CD133, aldehyde dehydrogenase 1 (ALDH1), CD9, ATP binding cassette subfamily C member 1 (ABCC1/MRP1), and connexin<br>- Enhanced cancer cell-killing and anti-tumor effects on cancer cells (mot-Adon)<br>- Enhanced apoptosis (mediated by reactivation of p53) (mot-Adon)<br>- Suppression of microvessel formation (mot-Adon)	[9, 16, 67, 70-75]

2
